# Associations of Physical Activity, School Safety, and Non-Prescription Steroid Use in Adolescents: A Structural Equation Modeling Approach

**DOI:** 10.3390/ijerph19010087

**Published:** 2021-12-22

**Authors:** Timothy A. Brusseau, Ryan D. Burns

**Affiliations:** Department of Health & Kinesiology, University of Utah, Salt Lake City, UT 84009, USA; ryan.d.burns@utah.edu

**Keywords:** bullying, gun violence, physical education, resistance training, schools, sports, weapons

## Abstract

Non-prescription steroid use can negatively impact adolescent physical and mental health and wellbeing. Determining correlates of this risk behavior is needed to help mitigate its prevalence. Two potential correlates are physical activity and school safety. The purpose of this study was to examine the associations of physical activity, school safety, and non-prescription steroid use within a sample of adolescents from the 2015–2019 US National Youth Risk Behavior Survey (YRBS). A multi-stage cluster sampling procedure yielded a representative sample of US adolescents from the 2015–2019 YRBS (*n* = 44,066; 49.6% female). Two latent variables indicating physical activity and unsafe schools were the independent variables. The dependent variable was a self-report of non-prescription steroid use. A weighted structural equation model examined the associations between physical activity and unsafe schools with non-prescription steroid use, controlling for age, sex, BMI %tile, race/ethnicity, and sexual minority status. The latent physical activity variable did not associate with non-prescription steroid use (β = 0.007, 95%CI: −0.01–0.02, *p* = 0.436); however, the unsafe schools latent variable did associate with non-prescription steroid use (β = 0.64, 95%CI: 0.59–0.69, *p* < 0.001). An unsafe school environment may be a determinant of non-prescription steroid use in adolescents. Physical activity behaviors did not associate with steroid use.

## 1. Introduction

Non-prescription steroid use is an adolescent risk behavior that may have both personal and environmental influences [1,2]. Anabolic-androgenic steroids are steroidal compounds that are chemically related to the sex hormone testosterone [1,2]. They are used in the clinical setting to stimulate muscle growth, increase appetite, stimulate male puberty, and treat chronic wasting conditions. [1,2] However, both athletes and non-athletes use steroids to improve muscularity, aggressiveness, and physical performance [1,2]. Like other unhealthy risk behaviors, adolescent steroid use may yield adverse consequences that outweigh potential gains that may delay or harm an adolescent’s development [3]. Adolescents may be more prone to risk taking behaviors compared to children and adults because of the surge of sex hormones during puberty onset that associates with sensation seeking and impulsivity [4]. Engaging in risk-taking behaviors activates the reward centers of the brain and it has been suggested that individual-level differences in reward drive may be a susceptibility factor to risk behaviors and make some adolescents more sensitive to the environment [5].

Not only have non-prescription steroids abuse have been prevalent in professional sports but they have recently have had a high prevalence in adolescent youth sports [6]. Competitive athletes wanting to increase their performance often abuse non-prescription steroids to improve workout recovery time, increase muscular strength, and increase fat-free mass [7]. Despite these performance-related benefits, adolescent non-prescription anabolic steroid use has been linked to damage to internal organs, arrest of bone growth, male feminization, female masculinization, and an increased risk of injection site infection [8]. Adolescent steroid use also correlates with risk behaviors including use of other illegal drugs, unprotected sex, and criminal behavior [9,10,11]. Although steroids can lead to an increase in athletic performance within the context of competitive sports, it is unclear if adolescents who participate in sports, resistance training activities, and other physical activities are truly more prone to take non-prescription anabolic steroids compared to non-physically active or lower physically active individuals, as some studies have shown a high prevalence of steroid use in non-athletes [12,13]. This suggests that non-athletes taking steroids may have either recreational (e.g., as part of a cluster of risk behaviors) or non-performance (e.g., physical appearance) related reasons for abuse outside of the physical activity/competitive sports context.

Schools play a major role in health behaviors and unhealthy risk behaviors in adolescents. School environments, or school climates, incorporates both the physical (e.g., appearance, organization, resources, comfort) and social (e.g., faculty-student interrelationships, equitable treatment of students, degree of social comparisons) aspects of a school [14]. A positive school environment yielding high levels of academic achievement can also lead to low levels of unhealthy risk behaviors [15]. Conversely, a negative school environment may facilitate unhealthy risk taking in adolescents [16]. A potential mechanism by which a negative school environment may influence risk behavior is through student perceptions of school safety, as it has been previous shown that facets of the school environment, including physical appearance and faculty-student interrelationships, associates with perceived school safety [14,17]. Additionally, unsafe school environments, especially students’ overall perceptions of their schools’ violence problems, have been associated with unhealthy risk behaviors in adolescents [18]. However, how high school students’ perceptions of school safety associates with non-prescription steroid use is unknown. How the presence of unsafe schools correlates with steroid use when also considering an adolescent’s physical activity behaviors is also unclear.

In the US, data from the 2015–2019 National Youth Risk Behavior Survey (YRBS) captures self-reported non-prescription steroid use, in addition to variables (items) related to physical activity and school safety [19,20]. Previous work using YRBS data has shown differential effects of non-prescription steroid use among different sexual minority and race/ethnicity groups within adolescents [21]. However, to our knowledge, no study has examined the independent associations of physical activity and school safety with non-prescription steroid use using recent YRBS data controlling for these demographic factors. Knowing this information can help derive preventive interventions by determining the behavioral and/or environmental influences of non-prescription steroid use in adolescents. Therefore, the purpose of this study was to examine the associations of physical activity, school safety, and non-prescription steroid use within a sample of adolescents from the 2015–2019 US YRBS.

## 2. Materials and Methods

### 2.1. Participants

Participants were a nationally representative sample of US high school students who were enrolled in the 9th–12th grades (*n* = 44,066; mean age = 16.0 ± 1.2 years old). Demographic characteristics are presented in Table 1. The sex distribution was approximately equal with a weighted female prevalence of 49.6%. The majority of the sample was White with a weighted prevalence of 53.1%. Approximately 69.4% of the sample was at a healthy weight with approximately 30.6% of the sample being either overweight or obese. The average body mass index percentile (BMI %tile) of the sample was 63.6 ± 28.7 based on self-report height and weight and the 2000 CDC BMI-for-age growth charts. Individual-level socioeconomic status data were not collected on the 2015–2019 US YRBS.

### 2.2. Instrumentation

**Figure 1:**
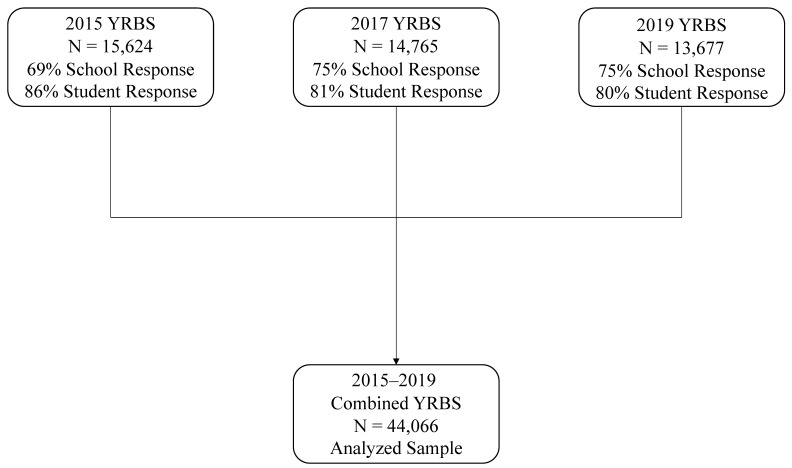
Flowchart showing the sample size, school response rate, and student response rate for the 2015, 2017, and 2019 US National Youth Risk Behavior Survey.

The United States Youth Risk Behavior Surveillance System YRBS is a data collection system established by the Centers for Disease Control and Prevention (CDC) in 1990. The 2015–2019 YRBS was a voluntary and anonymous self-report survey for high school students enrolled in US public and provite schools. The YRBS was administered throughout the US during the 2015, 2017, and 2019 spring semesters [22,23,24]. A multistage clustering sampling procedure was employed to recruit the participants Within the first stage, participating schools were selected systematically with the probability of selection proportional to grade 9–12 enrollment. In the second sampling stage, intact classes of a required subject or intact classes during a required period were selected randomly. All students in sampled classes were eligible to participate. A total of 182, 192, and 181 schools were sampled for the 2015, 2017, and 2019 YRBS respectively [22,23,24]. The school response rates were 69% in 2015 and 75% in both 2017 and 2019 [22,23,24]. The student response rates were 86% in 2015, 81% in 2017, and 80% in 2019 [22,23,24]. The overall response rate (school response rate × student response rate) was approximately 60% in 2015–2019 [22,23,24]. A study flowchart is provided within Figure 1. For each national YRBS dataset, a sampling weight based on student sex, race/ethnicity, and school grade was applied to each record to adjust for student nonresponse and the oversampling of Black and Hispanic students. The final overall weights were scaled so that the weighted counts of adolescents equaled the total sample size and the weighted proportions of adolescents in each grade matched national populations projections per survey year. The study was conducted according to the guidelines of the Declaration of Helsinki, and approved by the Institutional Review Board of the Centers for Disease Control and Prevention (protocol code #1969.0 and 11/10/15).

### 2.3. Measures

#### 2.3.1. Non-Prescription Steroid Use

Non-prescription steroid use was the observed dependent variable. Non-prescription steroid use was assessed using one item on the YRBS that asked, “During your life, how many times have you taken steroid pills or shots without a doctor’s prescription?”. The responses ranged from 0 times or 40 or more times using a 1–6 numerically coded response scale. The nonprescription steroid use dependent variable was analyzed on the continuous measurement scale.

#### 2.3.2. Physical Activity Items

Three YRBS items indicated the physical activity latent variable. One item asked, “During the past 7 days, on how many days were you physically active for a total of at least 60 minutes per day? (Add up all the time you spent in any kind of physicalactivity that increased your heart rate and made you breathe hard some of the time.)”. Responses ranged from 0 days to 7 days. Another item asked, “During the past 12 months, on how many sports teams did you play? (Count any teams run by your school or community groups.)”. Responses ranged from 0 teams to 3 or more teams. A final item asked, “During the past 7 days, on how many days did you do exercises to strengthen or tone your muscles, such as push- ups, sit-ups, or weight lifting?”. Responses ranged from 0 days to 7 days.

#### 2.3.3. Unsafe School Items

Three YRBS items indicated the unsafe school latent variable. One item asked, “During the past 12 months, how many times has someone threatened or injured you with a weapon such as a gun, knife, or club on school property?”. This item referred to other students either threatening or injuring the survey respondent and serves as an indicator for a safe school social environment. Responses ranged from 0 tiems to 12 or more times. Another item asked, “During the past 30 days, on how many days did you not go to school because you felt you would be unsafe at school or on your way to or from school?”. Responses ranged from 0 days to 6 or more days. A final item asked, “During the past 12 months, how many times were you in a physical fight on school property?”. Responses ranged from 0 times to 12 or more times.

#### 2.3.4. Demographic Covariates

**Figure 2:**
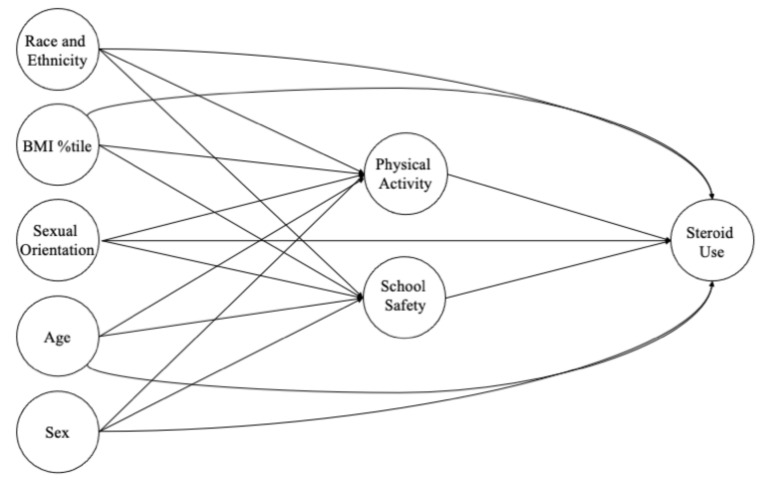
A directed acyclic graph (DAG) showing the theoretical and directional interrelationships among the study’s constructs.

Several observed demographic covariates were controlled for in the analysis. The covariates used in the analysis have all been shown to correlate with non-prescription steroid use, physical activity, and perceptions of school safety within previous studies. [1,2,3,4,5,6,7,8,9,10,11,12,13] A directed acyclic graph (DAG) was provided to show the interrelationships among the study’s constructs within Figure 2. Self-reported age was analyzed on the continuous measurement scale. Sex was a binary response item with males used as the referent level. Race/ethnicity was also controlled for in the analysis. The race/ethnicity variable was recoded to improve the low prevalence within specific race/ethnicity groups. A binary variable was derived for race/ethnic minority with White used as the referent level because of its high prevalence. A sexual minority variable was created from the self-report of sex and the sex of sexual partners. Sexual minority status was identified from either a self-reported same sex or both sex sexual partners; adolescents self-reporting opposite sex partners or no sex partners was the referent level for comparisons. Finally, BMI %tile was included as a covariate and was analyzed on the continuous measurement scale.

### 2.4. Statistical Analysis

The complex YRBS survey design, including the use of assigned strata, primary sampling units, and sampling weights was accounted for using Stata’s “svy:” prefix command to obtain valid variance estimates. Descriptive statistics were presented as means and standard deviations for continuous variables and as counts and weighted %’s for categorical variables. Differences between the sexes on the observed continuous variables were examined using independent t-tests. Effect sizes were calculated using Cohen’s delta (d) where d = 0.20 indicated a small effect size, d = 0.50 indicated a medium effect size, and d = 0.80 indicated a large effect size [25]. Differences between sexes on categorical variables were analyzed using chi-square tests.

The primary analysis involved the construction of a weighted structural equation model using Stata’s version 17.0 (Statacorp, College Station, TX, USA). “SEM Builder”. Latent variables for both physical activity and unsafe schools, both with three indicator variables, were associated with the observed non-prescription steroid use dependent variable. Covariances between latent variables were also constructed. The model was adjusted for age, sex, BMI %tile, race/ethnicity, and sexual minority status. Full information maximum likelihood was utilized, which has been shown to produce unbiased parameter estimates and standard errors in the presence of missing data [26]. Standardized path coefficients and covariances with 95% Confidence Intervals were reported. Unweighted overall model fit was determined using the Root Mean Square Error of Approximation (RMSEA; good fit ≤ 0.05) with *p*-close statistics (good fit > 0.05), and the Tucker-Lewis Index (TLI) and Comparative Fit Index (CFI; good fit ≥ 0.90) [27,28,29]. Weighted model fit was assessed using the coefficient of determination (R2).

## 3. Results

### 3.1. Descriptive Statistics

Approximately 2.8% of the sample reported ever using non-prescription steroids throughout their lifetime (2.5% of females vs. 3.6% of males; χ^2^ = 94.6, *p* < 0.001). Specifically, approximately 1.1% of the total sample reported using non-prescription steroids 1–2 times, 0.6% reported using 3–9 times, 0.3% reported using 10–19 times, 0.1% reported using 20–39 times, and 0.7% reported using 40 or more times. The adolescent response prevalence for the non-prescription steroid item was 36,282 out of the 44,066 total respondents (*n* missing = 7784). Descriptive statistics for the physical activity and unsafe school items are reported in Table 2. Males reported a higher weekly frequency of participating in 60 minutes of physical activity (d = 0.40, *p* < 0.001), participating in more sports teams throughout the past year (d = 0.20, *p* < 0.001), and more days per week of muscle strengthening activity (d = 0.44, *p* < 0.001) compared to females. Males also reported more times carrying a weapon at school (d = 0.16, *p* < 0.001) and being in more fights at school (d = 0.17, *p* < 0.001) compared to females.

### 3.2. Structural Equation Model Fit

**Figure 3:**
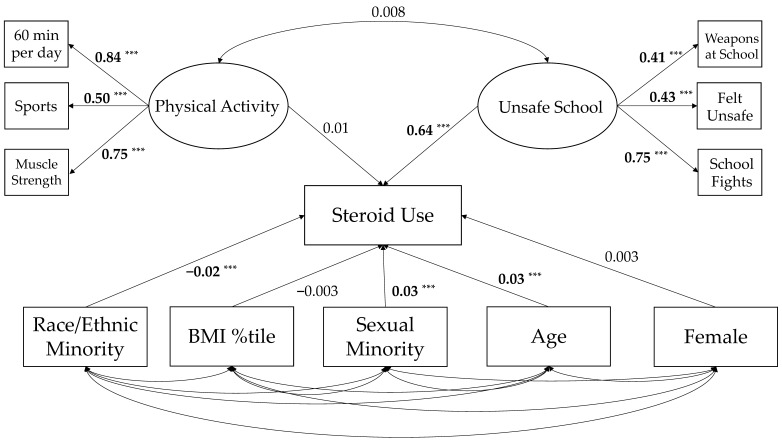
Weighted structural equation model showing the associations of physical activity, unsafe schools, and non-prescription steroid use, controlling for age, sex, BMI %tile, race/ethnicity, and sexual minority status. Note: Circles are latent variables and rectangles are observed variables; covariances between observed demographic variables and the latent factors are not displayed because of practical reasons but are reported within the text (Section 3.3) and within Table 3; bold denotes statistical significance, and *** *p* < 0.001.

The structural equation model is presented in Figure 3. The unweighted structural equation model yielded good overall model fit (RMSEA = 0.032, 90%CI: 0.030–0.033, *p*-close = 0.99; CFI = 0.951; TLI = 0.930). When sampling weights were applied to the model, the coefficient of determination was R^2^ = 0.94. Standardized covariances among the demographic variables are presented in Table 3.

### 3.3. Latent Variable Covariances

The standardized covariance between the two latent variables was not statistically significant (β = 0.008, 95%CI: −0.014–0.319, *p* = 0.470). Concerning the demographic covariates, age significantly inversely associated with unsafe schools (β = −0.07, 95%CI: −0.00–- 0.04, *p* < 0.001) and physical activity (β = −0.08, 95%CI: −0.10–−0.06, *p* < 0.001); BMI %tile significantly positively associated with unsafe schools (β = 0.04, 95%CI: 0.01–0.06, *p* = 0.002) and inversely associated with physical activity (β = −0.02, 95%CI: −0.03–0.00, *p* = 0.01); race/ethnic minority status positively associated with unsafe schools (β =−0.10, 95%CI: 0.08–0.10, *p* < 0.001) and inversely associated with physical activity (β = −0.10, 95%CI: −0.10–−0.08, *p* < 0.001); and being female inversely associated with unsafe schools (β = −0.11, 95%CI: −0.13–−0.09, *p* < 0.001) and physical activity (β = −0.26, 95%CI: −0.2 –−0.24, *p* < 0.001). Self-reporting as being a sexual minority inversely associated with physical activity (β = −0.10, 95%CI: −0.11–−0.09, *p* < 0.001) and positively associated with unsafe schools (β = 0.18, 95%CI: 0.17–0.20, *p* < 0.001).

### 3.4. Associations with Non-Prescription Steroid Use

The latent physical activity variable did not significantly associate with non-prescription steroid use (β = 0.007, 95%CI: −0.01–0.02, *p* = 0.436); however, the unsafe schools latent variable did significantly associate with non-prescription steroid use (β = 0.64, 95%CI: 0.59–0.69, *p* < 0.001). The demographic covariates that associated with non-prescription steroid use were adolescent age (β = 0.03, 95%CI: 0.02–0.05, *p* < 0.001), race/ethnic minority status (β = −0.02, 95%CI: −0.02–−0.01, *p* < 0.001), and sexual minority status (β = 0.03, 95%CI: 0.02–0.04, *p* < 0.001). Both BMI %tile (β = −0.003, 95%CI: −0.0–0.01, *p* = 0.653) and sex (β = 0.003, 95%CI: −0.010–0.010, *p* = 0.710) did not significantly associate with non-prescription steroid use.

## 4. Discussion

The purpose of this study was to examine the associations of physical activity, school safety, and non-prescription steroid use within a representative sample of US adolescents. The results indicated that perceived school safety associated with non-prescription steroid use while physical activity did not associate with non-prescription steroid use. The lack of an association between physical activity and non-prescription steroid use agrees with another large cross-sectional study that found that there was no correlation between steroid use and exercising behaviors when controlling for other substance use within a large sample of Norwegian adolescents [30]. These data highlight that important factors that may contribute to steroid abuse risk are factors other than being physically active, engaging in muscular strength exercises and/or participating in one or more sports teams. Additionally, using a large sample of 9–12th grade adolescents, Elkins et al. found that steroid use rates within a large US city was a function of specific school and parental factors, but not sports participation [1]. Past studies have shown adolescents who abuse non-prescription steroids also abuse other substances; therefore, steroid abuse in adolescents tends to be a part of a cluster of risk behaviors not necessarily tied to physical activity, sports participation, and athletic performance [31].

What is unclear and should be a priority for future research is if adolescent steroid abuse tracks into young adulthood. Previous work using cross-sectional research designs have showed that most of young adult male abuse of steroids did not start in adolescence but in young adulthood, with the motivations of improving muscularity and overall physical attractiveness [32]. Studies examining adolescent female steroid use however has shown great heterogeneity in prevalence estimates, showing prevalence as high as 7.3% and as low as 0.1%, possibly being a function of the phrasing of the questions within surveys and questionnaires [33]. Motivation for steroid use among females have been shown to include it being more of an impulsive choice, to overcoming fitness stagnation, and/or to improve self-protection abilities, especially after sexually traumatic experiences [34]. Longitudinal tracking of steroid abuse and motivation for abuse in both males in females is needed to help clarify its time varying aspects during different developmental stages and life transitions.

A second salient finding from the current analysis was that school safety associated with steroid use. This association was relatively strong in magnitude, even after controlling for several pertinent demographic covariates such as race/ethnicity and sexual minority status. Elkins et al. found that schools that frequently set and enforced rules regarding drug use tended to have lower rates of non-prescription steroid use [1]. Daily et al. found that schools that had a positive climate prevented substance-use initiation [35]. Because steroid use in adolescents tends to be a part of a cluster of risk taking and substance use behaviors, interventions targeting the school level may be an effective and efficient strategy to lower initiation of steroid abuse among high school students. Improving school physical appearance and accessibly to usable facilities, facilitating inclusiveness among faculty and students, improving the quality of faculty-student and student-student interrelationships, and making increasing the frequencies of messaging about positive and negative (risk) behaviors are all strategies to improve school climates [14,15,16,17,18]. However, what is unclear from the current study’s analysis is the direction of association between school safety and steroid use. Steroid use has been shown to correlate with aggressiveness and fights in youth [36,37]. Therefore, it is unclear if steroid use contributes to school safety or that if school safety contributes to steroid use, or both. Future research should test for reverse and bidirectional associations to better understand if school safety is indeed a determinant of steroid use risk within the adolescent population.

Strengths of this study include the use of a large and representative sample of adolescents, the use of several independent variables representing the constructs of physical activity and perceived school safety and the statistical control of several demographic variables that have previously shown to be determinants of non-prescription steroid use within the adolescent population. Limitations of this study include the use of a cross-sectional research design that precludes making any cause-and-effect inferences. All variables were collected using self-report methods that manifests the potential for recall and/or social desirability bias within the adolescents’ responses. Moderating and mediating variables were not tested but could be an analytic strategy for future research. There may have been a time lapse between responses to the items regarding physical activity (during the past week or the past 12 months) and the use of steroids at any time. Although, the model explained a significant portion of variation in non-prescription steroid use, there is still residual confounding factors not accounted for within the models. It is also important to consider the intentions and objectives for physical activity and sports participation, as adolescents participating in physical activity and sports for performance outcomes may have stronger correlations with the steroid outcome variable; however, these data were not collected on the US YRBS but should be considered in future research. Finally, the structural equation model was adjusted for several demographic covariates, but specific individual-level socioeconomic status data were not collected and may have confounded the observed associations.

## 5. Conclusions

Adolescent non-prescription steroid misuse continues to be a public health concern. This study showed that non-prescription steroid use is linked with perceived school safety but is unrelated to physical activity when controlling for pertinent demographic covariates such as race/ethnicity and sexual minority status. It is important for health educators and public health professionals to be mindful that adolescents who abuse non-prescription steroids may not necessarily be very active, participate in sports, and/or engage to higher amounts of strength training. Given the correlates observed in this study, derivation of multidimensional interventions targeting the school environment may be an effective approach to prevent adolescent non-prescription steroid use.

## Figures and Tables

**Table 1:** Sample demographic characteristics from the combined 2015–2019 Youth Risk Behavior Survey.

Variable	Level	*n*	Weighted %
Sex	Male	21,502	50.4%
	Female	22,168	49.6%
Race/Ethnicity	White	19,778	53.1%
	American Indian or Alaska Native	445	0.6%
	Asian	1893	4.1%
	Black or African American	6503	13.1%
	Native Hawaiian or Pacific Islander	285	0.6%
	Hispanic or Latino	4917	9.7%
	Multiple Races–Hispanic/Latino	6889	14.0%
	Multiple Races–Non-Hispanic/Latino	2223	4.8%
Weight Status	Healthy Weight	27,286	69.4%
	Overweight or Obese	12,358	30.6%
Sexual Minority	Non-Sexual Minority	33,711	93.2%
	Sexual Minority	2680	6.8%

**Table 2:** Descriptive statistics for the total sample and within sex specific groups (presented as means and standard deviations).

Latent Variable	Survey Item	Total Sample Mean (SD)	Females Mean (SD)	Males Mean (SD)
Physical Activity	Weekly Frequency of Meeting 60 minutes of Physical Activity (0–7 Scale) (*n* missing = 1363)	3.8 (2.5)	3.3 (2.4)	4.3 *** (2.5)
	Number of Sports Teams (0–3 Scale) (*n* missing = 9437)	0.9 (1.0)	0.8 (1.0)	1.0 *** (1.0)
	Weekly Frequency of Muscle Strengthening Activity (0–7 Scale) (*n* missing = 13,559)	2.8 (2.5)	2.2 (2.3)	3.4 *** (2.5)
Unsafe School	Carry a Weapon on School Property (0–4 Scale) (*n* missing = 1190)	0.1 (0.6)	0.05 (0.43)	0.15 *** (0.7)
	Absenteeism Because School is Unsafe (0–4 Scale) (*n* missing = 2731)	0.1 (0.6)	0.1 (0.5)	0.1 (0.5)
	Fight at School (0–7 Scale) (*n* missing = 1137)	0.1 (0.7)	0.1 (0.5)	0.2 *** (0.5)

Note: SD stands for standard deviation; bold denotes statistical differences between the sexes, *** *p* < 0.001.

**Table 3:** Standardized covariances among demographic variables from the weighted structural equation model.

	1. Age	2. Female	3. Sexual Minority	4. BMI %tile	5. Race/Ethnic Minority
1. Age	1				
2. Female	−0.03 ***	1			
3. Sexual Minority	−0.23 ***	0.11 ***	1		
4. BMI %tile	−0.04 ***	0.00	0.01	1	
5. Race/Ethnic Minority	0.01 *	−0.01	0.00	0.09 ***	1

Note: BMI stands for body mass index; bold denotes statistical significance, * *p* < 0.05, *** *p* < 0.001.

## Data Availability

The 2015, 2017, and 2019 Youth Risk Behavior Survey datasets are publicly available at the following website: https://www.cdc.gov/healthyyouth/data/yrbs/index.htm (accessed on 12 November 2021).
